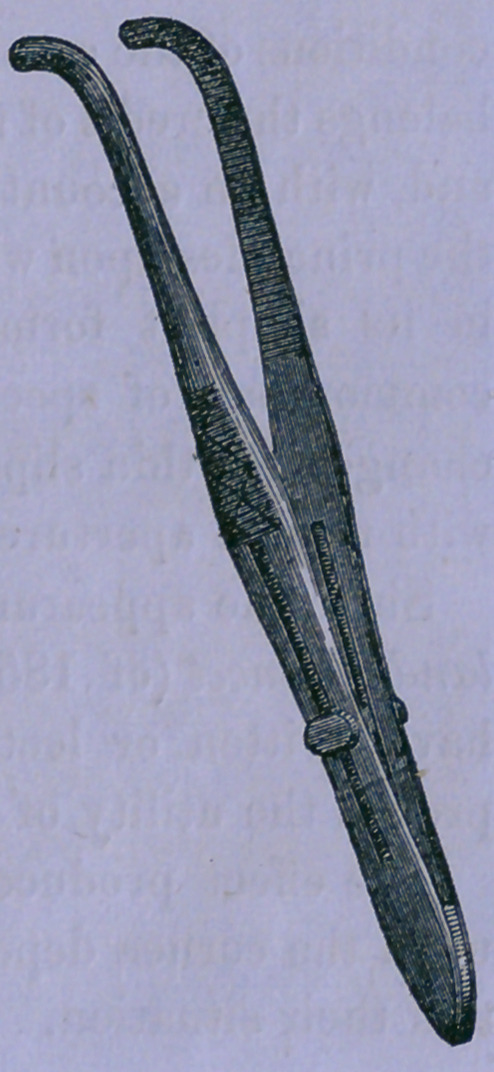# A New Method of Preventing Hemorrhage in Operations on the Lips

**Published:** 1859-08

**Authors:** Daniel Brainard

**Affiliations:** Surgeon of the U. S. Hospital at Chicago, etc.


ARTICLE III.

### A NEW METHOD OF PREVENTING HEMORRHAGE IN OPERATIONS ON THE LIPS.

BY DANIEL BRAINARD, M.D., SURGEON OF THE U.S. HOSPITAL AT CHICAGO, ETC.

Most surgeons will admit that hemorrhage is the most serious accident attending operations for hare-lip, and others of a similar character. This, it is true, does not, with care, often prove fatal, but it is hurtful in delicate children, and in all has the inconvenience from blood running into the throat, of rendering the use of chloroform dangerous. To prevent bleeding in operations on the lips, I have for many years been in the practice of directing an assistant to compress the facial artery with the fingers when it passes over the base of the lower jaw. The effect of this is only to diminish without arresting the hemorrhage. Compressing the whole cheek by passing a finger within the angle of the mouth, and passing it toward the thumb on the outside, is more effectual, but cannot be accomplished without interfering to some extent with the manipulations of the surgeon.

To avoid this, I have recently substituted common forceps for the finger, and embrace the upper lip on each side at a sufficient distance to allow the pins to be passed. This also is attended with some inconvenience, and I therefore had a small one constituted with a slide by which it is fixed. This is bent at the point, which should be turned toward the alæ nasi. This, with slight additional pressure by the fingers of an assistant at the side of the alæ nasi in cases of hare-lip, or upon the chin in operations for cancer of the lower lip, so effectually arrests the hemorrhage that chloroform may be safely used. These forceps are convenient for holding the lip while the needles are being inserted.

The figure represents the form of forceps which I have found useful, and is of half size. One should be applied on each side.

## Figures and Tables

**Figure f1:**